# Familial lichen amyloidosis as a presenting sign of multiple endocrine neoplasia type 2A tumor syndrome

**DOI:** 10.1016/j.jdcr.2026.08.001

**Published:** 2026-08-14

**Authors:** Caroline Readinger, Allison Readinger

**Affiliations:** a Texas A&M University, College of Biomedical Sciences, College Station, Texas; b Trinity Vista Dermatology, Fort Worth, Texas

**Keywords:** lichen amyloidosis, medullary thyroid carcinoma, MEN2A, pheochromocytoma, RET mutation

## Case report

A 26-year-old woman presented with a pruritic rash (Fig 1) on the upper portion of the back since childhood. Of note, her father had a similar rash (Fig 2) for many years. Biopsy showed rare apoptotic keratinocytes and lichenoid chronic inflammation. Re-review of the biopsy showed very focal deposits of keratin-derived amyloid in the papillary dermis (Fig 3).

**Fig. 1:**
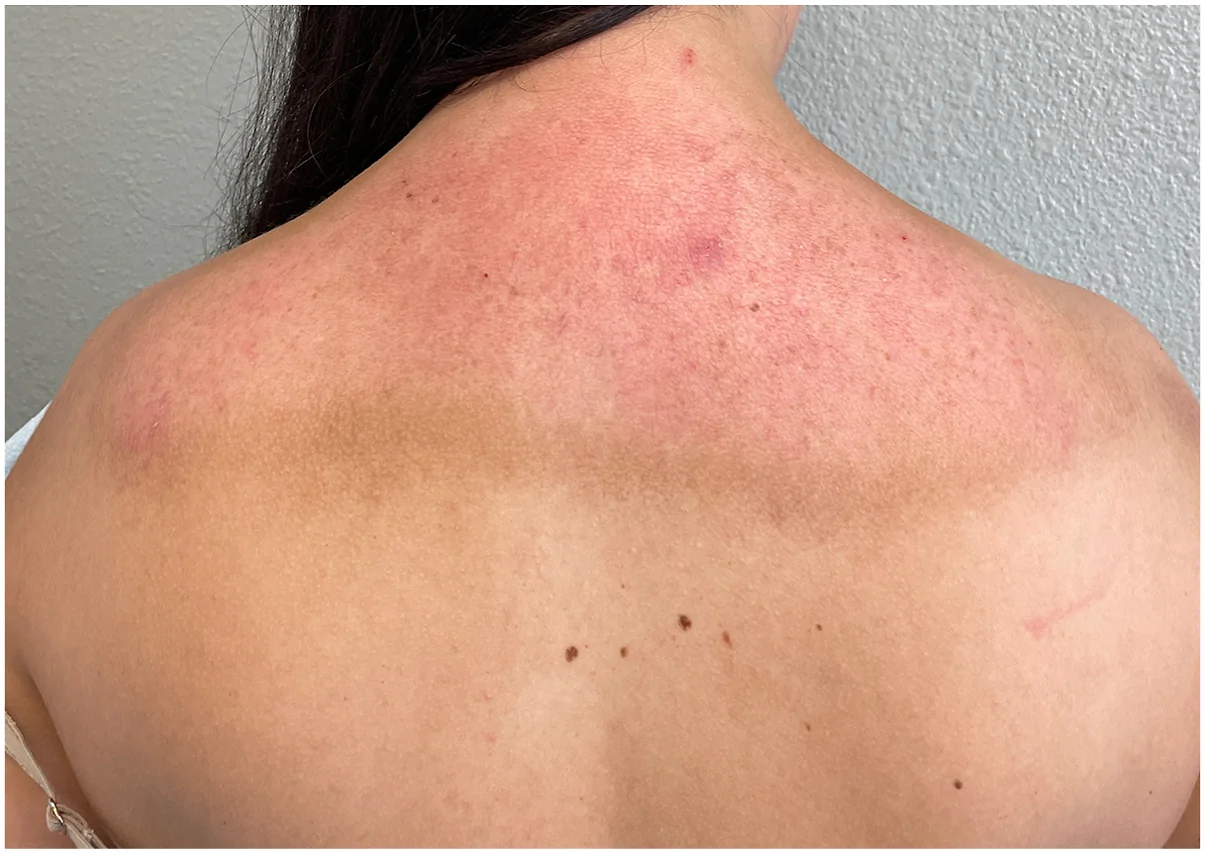
Patient with rash on upper portion of the back.

**Fig. 2:**
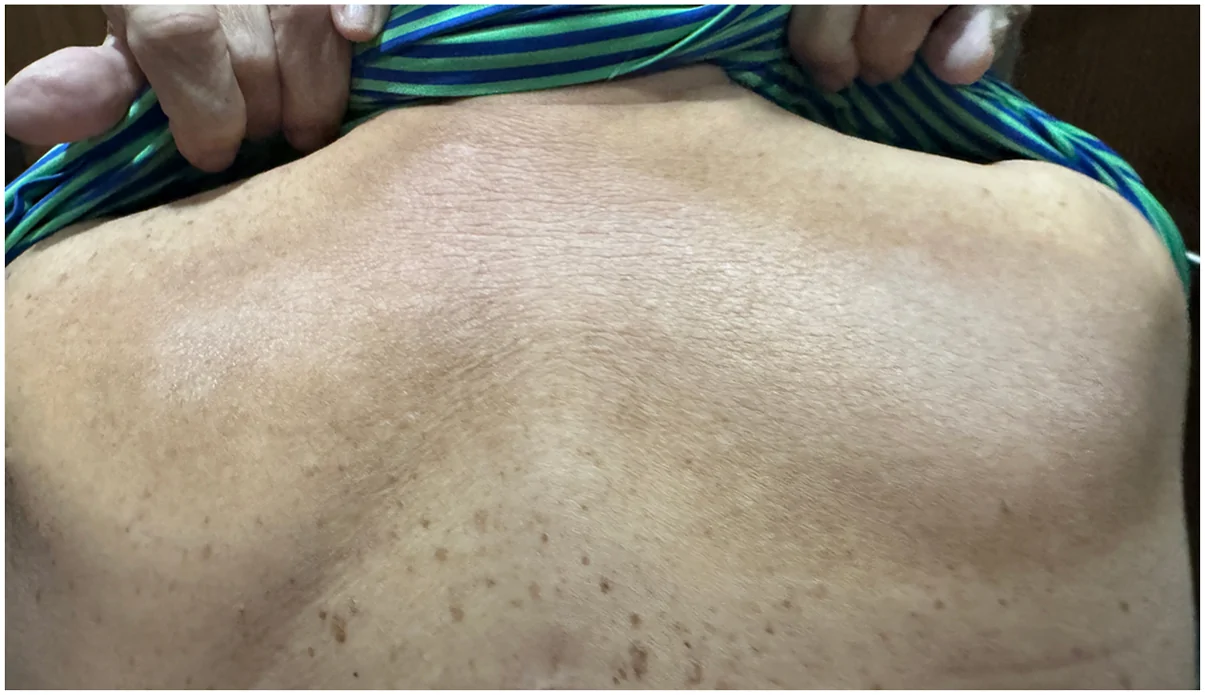
Patient’s father with similar rash on upper portion of the back.

**Fig. 3:**
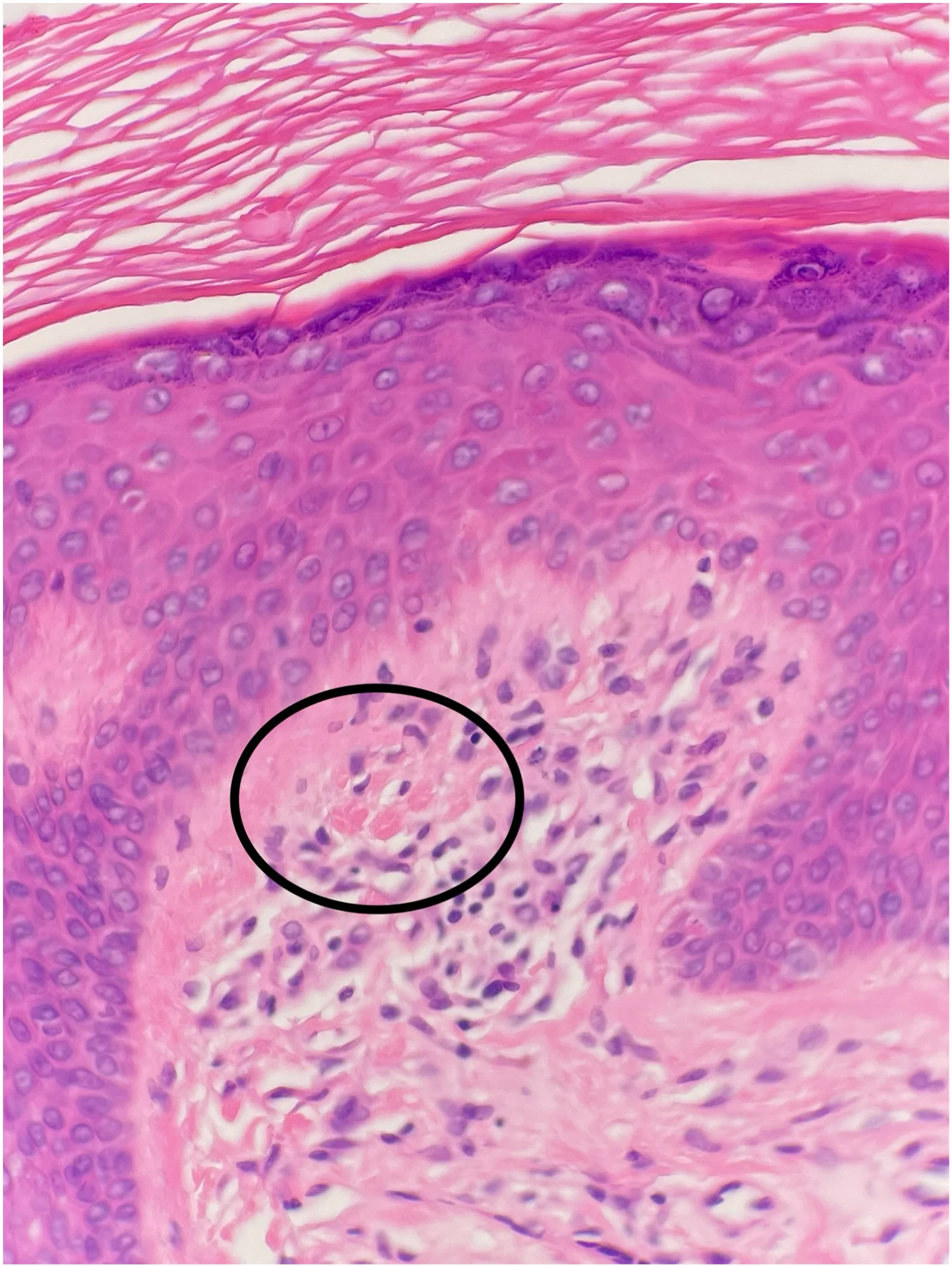
Biopsy (hematoxylin and eosin stain, high power) showing amyloid deposits (*circled*).

Question 1: What is a noncutaneous manifestation of this patient’s underlying hereditary syndrome?

A: Meningioma.

B: Leiomyoma.

C: Gastric adenocarcinoma.

D: Medullary thyroid carcinoma.

E: Melanoma.

**The correct answer is D:** medullary thyroid carcinoma.

Medullary thyroid carcinoma is a manifestation of multiple endocrine neoplasia type 2A (MEN2A) tumor syndrome. The patient was given a clinical diagnosis of lichen amyloidosis based on the cobblestoned appearance of the plaques. Intermittent treatment with topical steroids was initiated for symptomatic care, with instruction not to scratch the area. Repeat biopsy was considered at follow-up but not performed. Nine years later, the patient went to the emergency department with tachycardia and hypertensive crisis. Computed tomography scan showed an adrenal tumor, and pheochromocytoma (PHEO) was confirmed. A thyroid nodule, previously considered benign on ultrasound, showed medullary carcinoma on biopsy. Multiple endocrine neoplasia, type 2A, was confirmed by genetic testing, showing a mutation in the RET proto-oncogene. Her father was found to have the same mutation and was also diagnosed with a PHEO and medullary thyroid carcinoma. Her brother does not have the rash but does carry the RET gene mutation. He was also diagnosed with medullary thyroid carcinoma, PHEO, and parathyroid adenomas. A niece has the RET mutation and is being closely monitored, with plans for thyroidectomy before the age of 5 years. Lichen amyloidosis was the presenting sign of the MEN2A tumor syndrome in this family.

## Discussion

MEN2 variants are passed on in an autosomal dominant pattern and are caused by germline mutations in the RET proto-oncogene.^1^ MEN2A represents the classic, most common presentation, characterized by medullary thyroid carcinoma in 100% of affected individuals, with less frequent manifestations including PHEO in approximately 50% and primary hyperparathyroidism in about 15% of patients.^2^^,^^3^

Cutaneous lichen amyloidosis (CLA) is associated with MEN2A. The reported prevalence of CLA in patients with certain RET mutations ranges from 9% to as high as 50%.^2^^,^^4^ When CLA presents as the initial clinical feature, it can serve as an early indicator of underlying MEN2A. Once an RET mutation is identified in a patient, cascade screening of relatives becomes essential, even in the absence of CLA. Tumor surveillance can be initiated, and prophylactic thyroidectomy may be performed.^3^ Specifically, in individuals known to have the RET C634 mutation, thyroid removal is recommended at approximately 5 years of age or earlier if serum calcitonin levels are elevated.^3^ Awareness of the association of CLA with MEN2A is essential to early recognition of the tumor syndrome. Patients with similar findings, particularly those with lichen amyloidosis at an early age, should be questioned about family history, and genetic screening should be considered.

## Conflicts of interest

None disclosed.
